# A modular technique of Booth encoding and Vedic multiplier for low-area and high-speed applications

**DOI:** 10.1038/s41598-023-49913-5

**Published:** 2023-12-16

**Authors:** C. M. Kalaiselvi, R. S. Sabeenian

**Affiliations:** 1 Sona College of Technology, Salem, India; 2 Department of ECE, Sona College of Technology, Salem, India

**Keywords:** Engineering, Mathematics and computing

## Abstract

A technique for efficiently multiplying two signed numbers using limited area and high speed is presented in this paper. This work uses both the Booth and Vedic multiplication sutra methodologies to enhance the speed and reduction in the area by using two VLSI architectures of radix encoding techniques—Radix-4 and Radix-8—with the Vedic multiplier. The functionality of the proposed methods is tested using an Artix-7 Field Programmable Gate Array (FPGA-XC7A100T-CSG324) in Xilinx Vivado 2019.1 and ASIC 45 nm technology. Two methods of Booth encoding using Vedic multiplier (Urdhva-Tiryakbhyam sutra) were used to develop, and examine the benefits of rapid computational multiplier. The results of the proposed multiplier for Booth-Vedic-Radix-4 encoding (BVR-4) decrease area by 89% and improve Area-Delay Product (ADP) by 72% for a 16-bit multiplier when subjected to other existing multipliers. The Booth-Vedic-Radix-8 (BVR-8) method shows that there will be an 89% reduction in area and an improvement in ADP by 72% for the 16-bit multiplier. The performance is evaluated regarding area occupancy (i.e., LUTs number) and propagation delay (output time). In terms of resource utilization, the proposed BVR-4 and BVR-8 multipliers outperform all the current designs with a marginal effect on speed and area for narrower bit-width ranges.

## Introduction

Everyone nowadays wants portable technology that is quick, takes little power, and packs as many components as possible into a small space. To achieve all of these goals, it is critical to develop an algorithm that solves all of these challenges. Although these methods are fast, the actual calculation time is determined by the processor’s system clock^1^. In processors, Multiplication is now an essential component of binary computers. Therefore, building multipliers is critical for computer scientists and engineers on both theoretical and practical levels^2^. Multipliers can generally be built in parallel or serial fashion. Serial multipliers are crucial when contemplating low-cost designs. Parallel multipliers are the preferred solution if a high-speed architecture is required. Furthermore, multipliers can be both signed and unsigned^3^.

## Related work

The prevalent multiplication procedure consists of three major phases:

1. Partial product generation (PPG)—Reduction of bitlength.
2. Partial product reduction (PPR)—Vertical processing.
3. Partial product accumulation (PPA)—Addition.

The second phase is critical for power consumption, cost, and overall performance. As a result, the power consumption and performance constraints of the multiplier are essential^4^. The add-and-shift operation of the array multiplier produces more partial products (PP) than the tree multiplier, boosting Power and Delay^5^. Despite having fewer components than an array multiplier, the PPs are organized in rows or columns and have extensive interconnections^6^. Dadda Multiplier has a higher carry propagation adder since it just makes the necessary reduction. Dadda Multiplier outperforms Wallace Multiplier in terms of speed^7^. The Karatsuba algorithm performs quick multiplication. It employs a smaller number of little multipliers than the Wallace tree multiplier^8^. To do the multiplication, a fast Carry-Save Add-shift (CSAs) multiplier is employed, which results in one-third the speed of an unsigned multiplier^9^. A fast multiplication in 2’s complement format is described to create fewer PP rows from n/2 + 1 to n/2 before applying the PPR approach to prevent the occurrence of the additional row, which increases multiplication speed for 8-bit and 16-bit multipliers^10^.

Using one of the several multiplication algorithms available, the speed of operation can be boosted over the classic shift and add approach. Booth’s method, a uniform shift approach, examines two multiplier bits at a time to identify the correct multiplicand multiple to add the PP^11^. Carry-free addition is accomplished by the use of redundant binary systems. The propagation delay problem is solved by the Redundant Binary adder^12^. For some signed numbers-based applications, it may still be viable to develop the requisite hardware accelerators utilizing unsigned multiplier designs^13^.

The number of PPs is proportional to the Radix-k of Booth encoding by a factor of log_2_ (k), implying that the number of PPs reduces by half while Radix-k multiplies by four^14^. The Radix-k Booth encoding system is identified by the equation

1 $${\text{Radix-k}}=1+{\text{log}}2\mathrm{ r},$$

where r is the number of encoding bits to be considered^15^. For example, Radix-4 Modified Booth Encoding (MBE) requires multiples of the multiplicand (X) of 0, 1, and 2 to generate PPs, lowering the height of the PP matrix from N to N/2. All of these multiples are easily obtained by using shift and negation operations on X. Similarly, Radix-8 MBE requires X multiples of 0, 1, 2, 3, and 4, while lowering the height of the PP matrix from N to N/3^16^. The redundant binary PPG achieves the largest reduction in the number of PPs, around 75%, for a Radix-4 multiplier^17^.

A modified Radix-4 Booth multiplier that only adds non-zero Booth encodings and disregards zero operations^18^. To do the hard multiple 3X operations, use 3X = 2X + X^19^. Radix-8 Booth multiplier uses the approximation approach by employing an approximate 2-bit adder^20^. There are two types of modular hybrid adder architectures employed. It is created by combining carry-skip, carry look-ahead, and Ripple Carry Adder (RCA)^21^. The high-performance Redundant binary multiplier’s appeal has been largely attributed to two key features: high modularity and carry-free addition^22^. Table 1 summarizes all of the multipliers and displays their properties in terms of Area, Power, Complexity, Delay, and Implementation.

**Table 1:** Multipliers circuit comparison^14^.

S. no.	Parameters	Shift and add	Array multiplier	Modified booth multiplier	Modified booth wallace multiplier	Vedic multiplier
1.	Serial/parallel	Serial	Parallel	Parallel	Parallel	Parallel
2.	Area	Small	Large	Medium	Medium	Medium
3.	Power consumption	Small	Large	Medium	Medium	Medium
4.	Delay	Large	Medium	Small	Smallest	Smallest
5.	Complexity	Simple	Simple	Complex	Complex	Simple
6.	Implementation	Easy	Easy	Medium	Difficult	Medium

A multi-precision binary multiplier architecture was created to reduce hardware and space consumption as well as latency and delay^23^. The beginning of the twentieth century saw the deciphering of the incredible Vedic calculation method. The functionality of Vedic mathematics is its most astounding feature^24^. Table 2 illustrates the strategies and procedures that Tirthaji created to reinforce the rules found in 16 sutras and 13 up-sutras, which were called Vedic Mathematics. The Nikhilam and Urdhva sutras were the focus of most investigations for the development of multipliers^25^. Researchers have also employed Nikhilam and UT multiplication methods. Due to the simultaneous creation and addition of the PPs, the former approach is quick^5^. Beneficial takeaways from both sutras have been made, and a novel architecture for quick and effective multiplication has been constructed^26^.

A multiplier architecture is designed for low-cost power and high-performance applications based on ancient Vedic Mathematics^27^. Vedic methods were used to square, and the results were evaluated using Virtex4vlx15sf363-12^28^. The 8 × 8 Nikhilam sutra is actualized for three different sets of bases using the idea of UT^29^. For a 16-bit multiplier, a novel Vedic Mathematics architecture built on UT was introduced. To lower the vertical critical route delay, compressors are utilized with full and half adders^30^. The Vedic multiplier is a combination of two compressor types: 4:2 and 7:2. After calculating the area and latency, it was shown to be 1.12 times faster than the other multiplier^31^.

**Table 2:** Sixteen Vedic sutras^32^.

S. no.	Name	Meaning
1.	Anurupyeshunyamanyat	If one is in ratio, the other is zero
2.	ChalanaKalanabyham	Similarities and differences
3.	Ekadikena Purvena	Compared to the previous one, it is one more
4.	Ekayunene Purvena	Compared to the previous one, it is one less
5.	Gunakasamuchyah	The sum of the factors = factor of the sum
6.	Gunitasmuchyah	The sum of the product = product of the sum
7.	Nikhil Navatashcaramam Dashtah	All from 9 and the last from 10
8.	Paraavartya Yojayet	Adjust and transpose
9.	Puranapuranabhyam	By non-completion or completion
10.	Sankalana Vyavakalanabhyam	By subtraction and addition
11.	Shesanyankena Charamena	The remainder by the last digit
12.	Sopaantyadvayamantyam	The ultimate and twice the penultimate
13.	ShunyamSaamyasamuccaye	If the sum same and that sum is zero
14.	Urdhva Tiryakbhyam	Cross-wise and vertically
15.	Vyashtisamanstih	Whole and part
16.	Yaavadunam	Whatever the extent of its deficiency

The redundant binary representation of the Signed Vedic multiplier (SVM) architecture was used in the UT sutra. It is possible to achieve carry-free addition in redundant binary representation^33^. The proposed method performs multiplication using a combination of the UT algorithm and Booth encoding. The multiplier is small and requires less space based on Booth encoding, but the UT sutra increases speed^34^. Based on the anurupyena sutra of Vedic arithmetic, it uses a high-performance and area-efficient square architecture for variable bit operands^35^. The Spartan 6 FPGA implementation models the hybrid parallel adder-based multiplier, which is utilized to improve multiplier performance^36^.

One drawback of the current technique is that it takes up more space and has a bigger delay in the PPR (second stage of the multiplication phase)^25,33,34^. All previously substantiated Vedic systems can handle only unsigned integers, which summarises the complexity of the proposed technique. By using the Booth encoding process and the UT technique of the Vedic multiplier in the second step, we have expanded the applicability of Vedic Mathematics to signed integers in this work. The signed Vedic multiplier and Booth encoding both resolve the carry-free propagation issue, which is the motivating factor behind the suggested work. The distinctions between the Vedic multiplier and the Booth multiplier have been examined in several survey articles. However, in this paper, a novel efficient architecture for quick multiplication computation is developed by combining the Booth encoding and Vedic multiplier techniques due to the advantages of parallel processing at the PPR stage and by taking into account the key advantages of all the sutras^33,37–42^. Removal of noise in digital images is done through the filter^43^. Less heat dissipation with reversible logic gates was designed^44^.

The contributions of this paper are as follows:

1. This work mainly focuses on the second stage of PPR since it consumes more area with less speed. Recent research focuses on PPR to reduce the area and improve the speed performance. This research focuses on PPR and how Booth encoding methods and Vedic sutras reduce area while improving speed. In the proposed study, both techniques combine the benefits of high speed with the method of Vedic sutras and low area with Booth encoding.
2. The reduction of propagation delay in the PPR using a novel encoder is proposed. The proposed method extends the Vedic architecture to signed integers.
3. Fewer resources were used when the Booth multiplier and Vedic multiplier (UT) sutra were combined.

The section of the paper is structured as follows: The proposed method for the BVR-4 and BVR-8 systems is covered in “Proposed method” section. The findings and a discussion of the suggested BVR-4 and BVR-8 design are given in “Results and discussion” section. In “Conclusion” section, the paper concludes.

## Proposed method

Effective computing units are provided by the Vedic design in the review of existing methods, but only for unsigned values. When thoroughly examined, signed numbers are generally the most appropriate for a variety of purposes. The proposed method expands the applicability of Vedic architecture to signed integers and offers a reduction in area and time-efficient architecture at the PPR level. The recommended method is divided into three stages overall.

First, Booth encoding (multiplier trits are encoded) is performed.

(i) PPG-reduction of Bit length
(ii) PPR-vertical processing
(iii) PPA- addition

### Stage-I: PPG-booth encoding

#### Radix-4 booth algorithm

The first section discusses how the Radix-4 encoding rule works. A normal multiplication procedure must be used to multiply the multiplicand and multiplier of n bits, which results in n-rows of partial products and an increase in time complexity and area. Comparing the modified Booth multiplier-Radix-4 to other types of serial-parallel multipliers, it is smaller, quicker, and uses less power. An n-bit multiplication operation requires less space when using the Radix-4 Booth encoding approach since only half of the PP rows are required.

**Figure 1:**
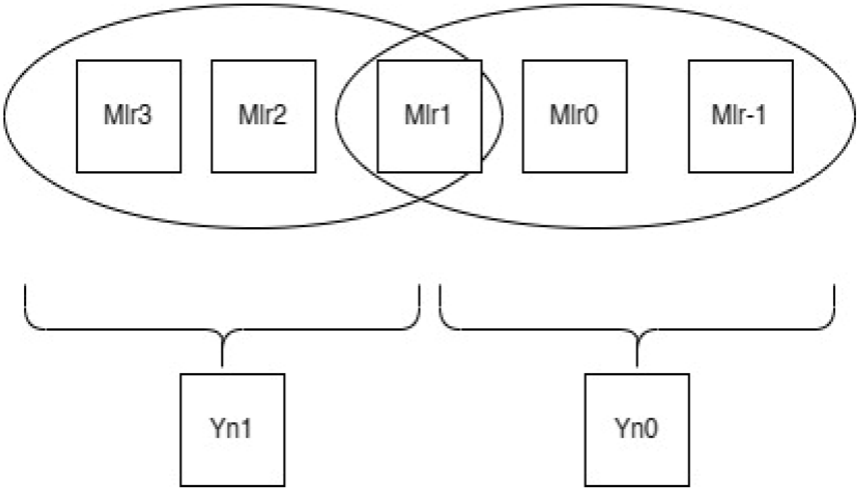
Contiguous trits of multiplier-4-bit width. *Mlr* multiplier bits, *Yn0, Yn1* new encoding groups of 1 and 2.

**Figure 2:**
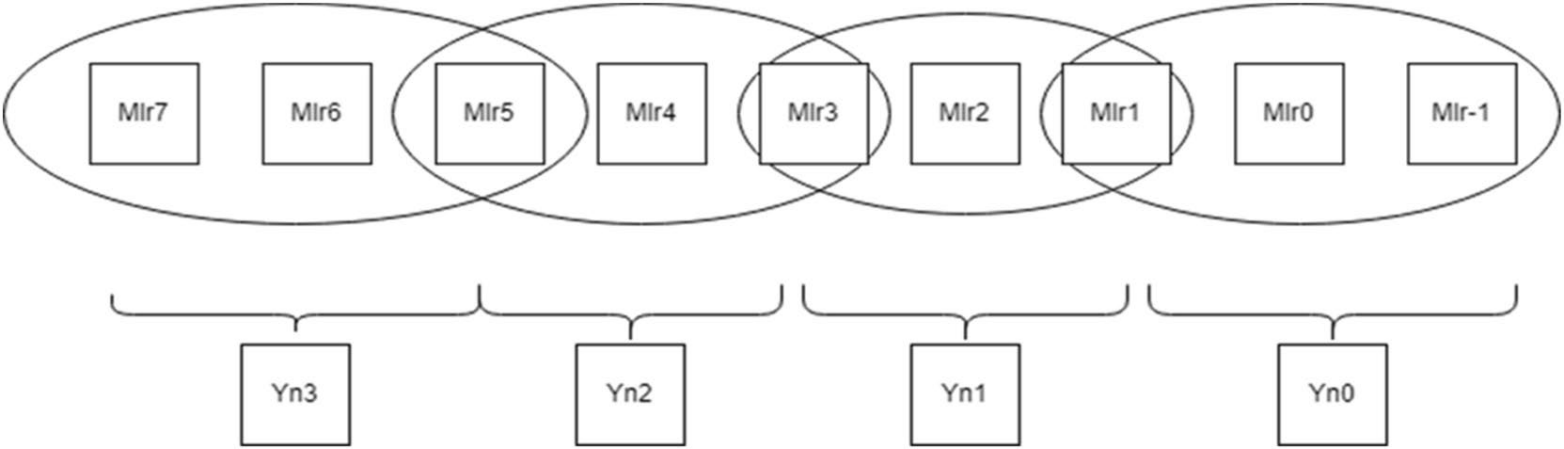
Contiguous trits of multiplier—8-bit width. *Mlr* multiplier bits, *Yn0, Yn1, Yn2, Yn3* new encoding groups of 1, 2, 3 and 4.

**Figure 3:**
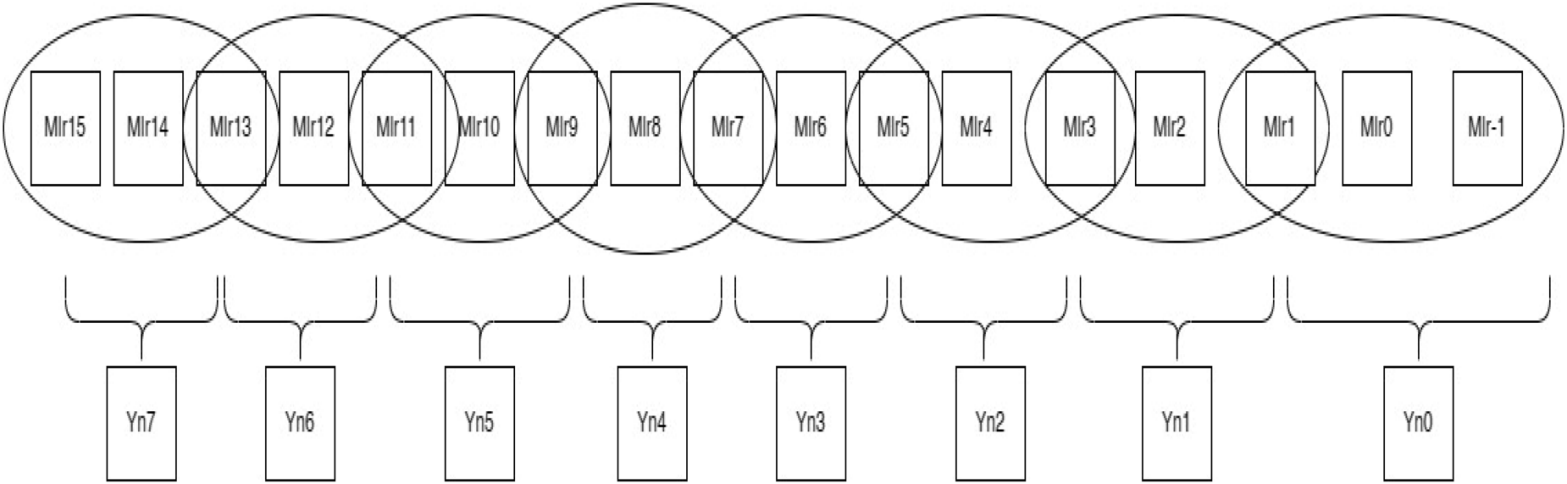
Contiguous trits of multiplier—16-bit width. *Mlr* multiplier bits, *Yn0, Yn1, Yn2, Yn3, Yn4, Yn5, Yn6 and Yn7* new encoding groups of 1, 2, 3, 4, 5, 6, 7 and 8.

As seen in Fig. 1, the contiguous multiplier trits are clustered together with the overlapping of the last bit in the past cluster to generate new encoding groups. For instance, a multiplier bit-width of 4 results in the creation of two new Yn0 and Yn1 encoding groups, which lowers the total PP row to half of its original rows. In the same way, Fig. 2 bit-width of 8 results in the formation of four new encoding groups for Yn0, Yn1, Yn2, and Yn3, and Fig. 3 bit-width of 16 results in the formation of eight new encoding groups for Yn0, Yn1, Yn2, Yn3, Yn4, Yn5, Yn6, and Yn7. However, when compared to the conventional multiplication procedure, the number of PP rows will be greater when compared with the Booth encoding procedure. This is the main benefit of the proposed method in the initial PPG step. The PP rows were reduced to half the number of varying operand sizes.

To calculate the PP rows for the operand size of

1. 4—The number of rows required is 2.
2. 8—The number of rows required is 4.
3. 16—The number of rows required is 8.

For the proposed method of BVR-4, the pseudo-code is explained below. The given data is first encoded according to Table 3, and then the multiplication operation is carried out.

**Table 3:** Radix-4 encoding rule.

S.no.	Multiplier bits			Encoding rule	Resultant encoded bits		
	Mlr_2_	Mlr_1_	Mlr_0_		R2	R1	R0
1.	0	0	0	0	0	0	0
2.	0	0	1	+ 1	0	0	1
3.	0	1	0	+ 1	0	0	1
4.	0	1	1	+ 2	0	1	0
5.	1	0	0	− 2	1	1	0
6.	1	0	1	− 1	1	1	1
7.	1	1	0	− 1	1	1	1
8.	1	1	1	0	0	0	0

##### Rule for combining adjacent trits


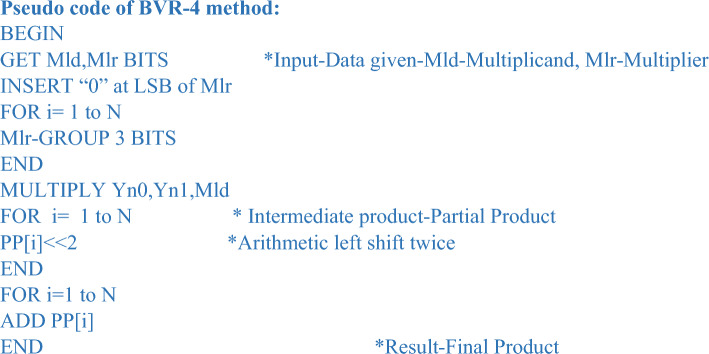

1. To check whether the given bit-width is odd or even if it is odd perform a sign extension of 1 bit and if it is even no need to perform a sign extension.
2. The foremost step is to add “0” at the LSB of the multiplier bits.
3. Further, the successive clustering of trits is carried out.
4. New encoding groups are created to perform the multiplication operation.

In Table 3, the multiplier trits are encoded using the Radix-4 encoding rule to build a new encoding group in the form of a two’s complement representation of the multiplier trits spanning from 0, + 1, + 2, − 2, − 1. The multiplier bits are multiplied with the multiplicand to accomplish the PPG operation, which results in the PP rows, according to the new encoded rule. The following stage of the operation was carried out after the production of incomplete product rows. Following the encoding procedure, the encoded bits create new groups of trits, each of which is represented in non-redundant radix-4 format.

#### Radix-8 Booth algorithm

A higher representation radix results in fewer digits when representing a given range of integers. As a result, as we advance to higher radices, a digit-at-a-time multiplication method uses fewer cycles. The Radix-8 Booth method decreases the PP rows even more to [(N/3) + 1], resulting in a substantially smaller area utilized when compared to the Radix-4 approach. The multiple hard problems are solved in this approach by expressing the 2’s complement of multiplier bits and multiplying it with the multiplicand bits.

**Figure 4:**
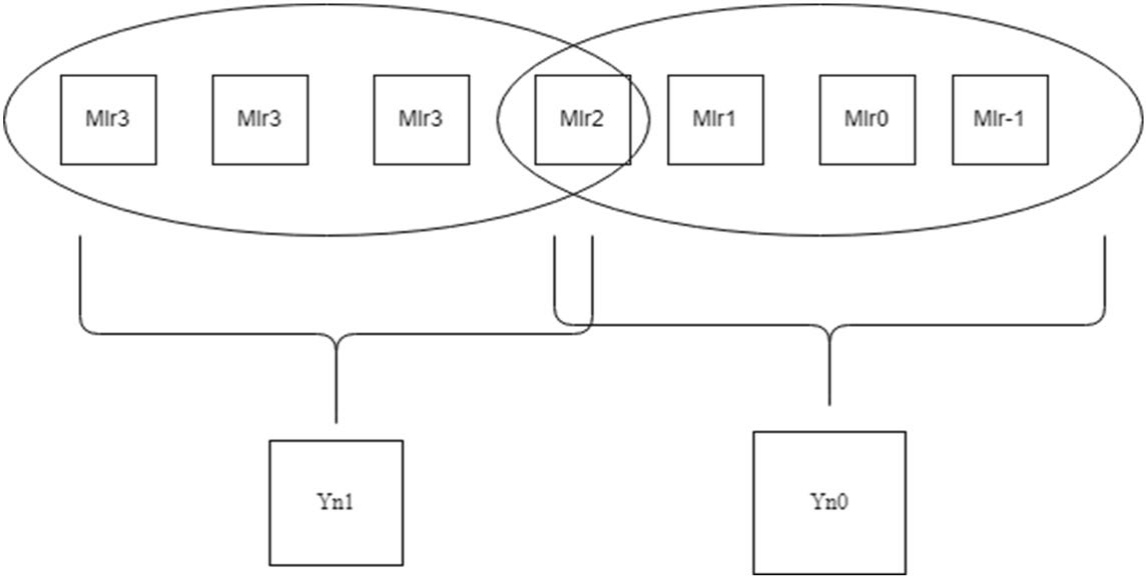
Contiguous quads of multiplier-4 bit-width. *Mlr* multiplier bits, *Yn0, Yn1* new encoding groups of 1 and 2.

**Figure 5:**
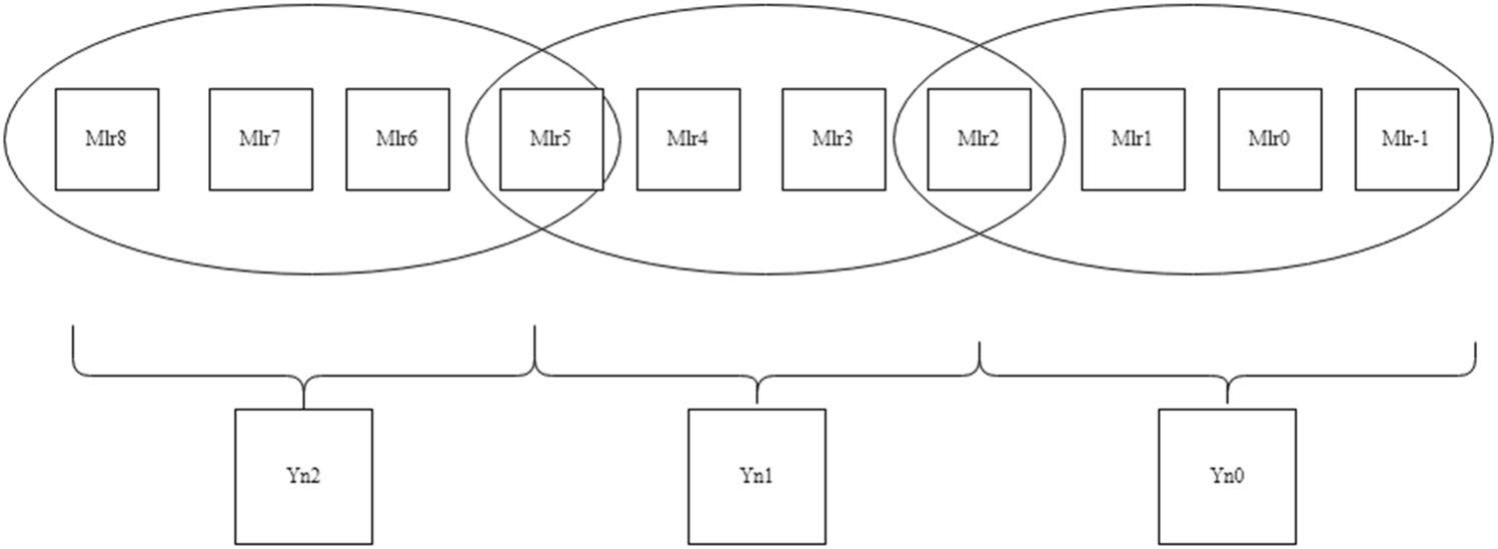
Contiguous quads of multiplier—8 bit-width. *Mlr* multiplier bits, *Yn0, Yn1, Yn2* new encoding groups of 1, 2 and 3.

**Figure 6:**
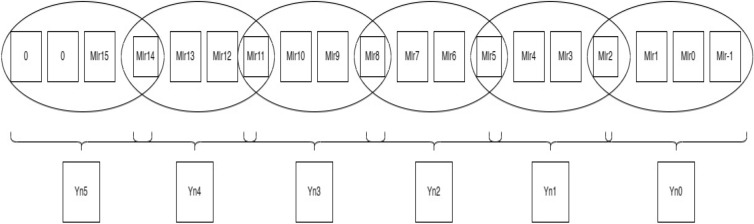
Contiguous quads of multiplier—16 bit-width. *Mlr* multiplier bits, *Yn0, Yn1, Yn2, Yn3, Yn4 and Yn5* new encoding groups of 1, 2, 3, 4, 5 and 6.

In Fig. 4, multiplier bits are grouped in terms of the quad to form a new encoding group, for example, a multiplier bit-width of 4 creates 2 new encoding groups with the sign extension of multiplier bits for lack of contiguous quads, thereby reducing the overall PP row into half of their original rows. Similarly, in Fig. 5, for a bit-width of 8, it forms a 3 new encoding group, and similarly, in Fig. 6, for a bit width of 16, it forms a six new encoding group. So, when compared with the Radix-4 encoding rule it produces fewer PP rows. To calculate the PP rows for the operand size of

1. 4—The number of rows required is 2.
2. 8—The number of rows required is 3.
3. 16—The number of rows required is 6.

##### Rule for combining adjacent quads


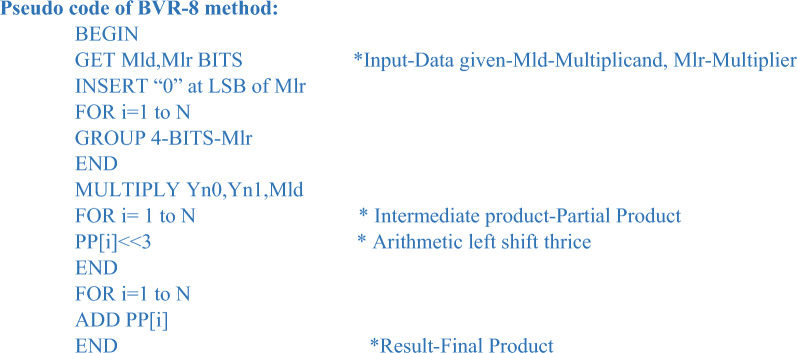

1. To check whether the given bit-width is odd or even if it is odd perform a sign extension of a sufficient bit and if it is even no need to perform a sign extension.
2. The foremost step is to add “0” at the LSB of the multiplier bits.
3. After adding, the successive grouping of the quad is carried out.
4. New encoding groups are created to perform the multiplication operation.

For the proposed method of BVR-8, the pseudo-code is explained below. The given data is first encoded according to Table 4, and then the multiplication operation is carried out.

**Table 4:** Radix-8 encoding rule.

S.no.	Multiplier bits				Encoding rule	Resultant encoded bits			
	Mlr_3_	Mlr_2_	Mlr_1_	Mlr_0_		R3	R2	R1	R0
1.	0	0	0	0	0	0	0	0	0
2.	0	0	0	1	+ 1	0	0	0	1
3.	0	0	1	0	+ 1	0	0	0	1
4.	0	0	1	1	+ 2	0	0	1	0
5.	0	1	0	0	+ 2	0	0	1	0
6.	0	1	0	1	+ 3	0	0	1	1
7.	0	1	1	0	+ 3	0	0	1	1
8.	0	1	1	1	+ 4	0	1	0	0
9.	1	0	0	0	− 4	1	1	0	0
10.	1	0	0	1	− 3	1	1	0	1
11.	1	0	1	0	− 3	1	1	0	1
12.	1	0	1	1	− 2	1	1	1	0
13.	1	1	0	0	− 2	1	1	1	0
14.	1	1	0	1	− 1	1	1	1	1
15.	1	1	1	0	− 1	1	1	1	1
16.	1	1	1	1	0	0	0	0	0

In Table 4, the multiplier quads are encoded using the Radix-8 encoding rule to build a new encoding group in the form of a two’s complement representation of the multiplier. The multiplier bits are multiplied with the multiplicand to accomplish the PPG operation, which results in the PP rows, according to the new encoded rule. The following stage of the operation was carried out after the production of incomplete product rows. Following the encoding procedure, the encoded bits create new groups of quads, each of which is represented in non-redundant Radix-8 format.

### Stage-II: PPR (Vedic multiplication)

Because PPG and PPR occur serially in a typical Booth multiplier, the vertical critical path latency increases. As a result, as a unique feature introduced in this research, the produced PPs are achieved by parallel processing in the second stage of PPR. This is one of the benefits of the unique work that was carried out. Because the second stage consumes the majority of the space, power, and delay in all assessed papers, an innovation was added in the second stage to carry out the PPR approach by employing the notion of Vedic Mathematics. Among the sixteen sutras in Vedic Mathematics, the UT and Nikhilam sutras are ideally suited for multiplication. The former sutra is usable for both binary and decimal multiplication and is best suited for lesser bit widths, whereas the latter sutra is best suited for greater bit widths multiplication. The PPR stage in the proposed work was carried out utilizing the Vedic multiplier idea employing the UT sutra (criss-cross multiplication), which involves its operation by simply conducting the AND gate operation in all bit multiplication procedures.

The newly encoded groups conduct vertical and cross-wise multiplication using the UT sutra. The resulting PPs are formed after conducting the criss-cross procedure. Similarly, the same method is used for operand sizes with varying bit widths.

Furthermore, by employing the UT sutra, there is no need to wait for all PP generations to mature before obtaining the final output. Among the 16 sutras, the UT sutra was chosen specifically because it is suitable for both decimal and binary multiplication. It has a basic construction since it just uses AND gates, half adders, and full adders. Following the multiplication through the AND gate, each PP element is received in parallel, and the final result is achieved.

**Figure 7:**
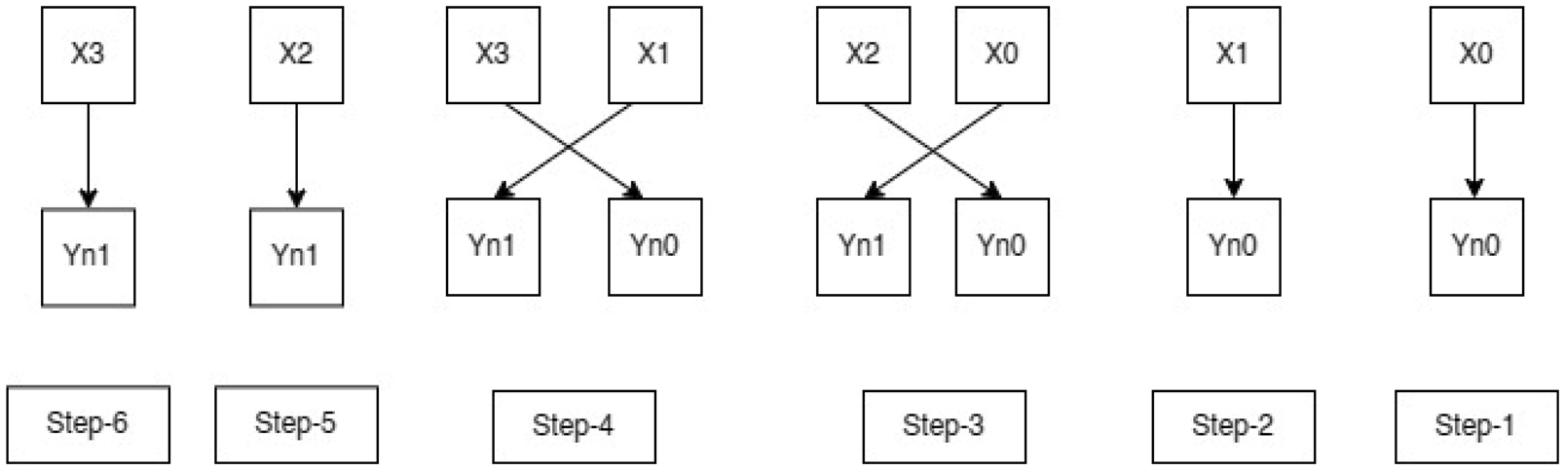
Vedic multiplication of 4-bit-width using criss-cross sutra.

**Figure 8:**
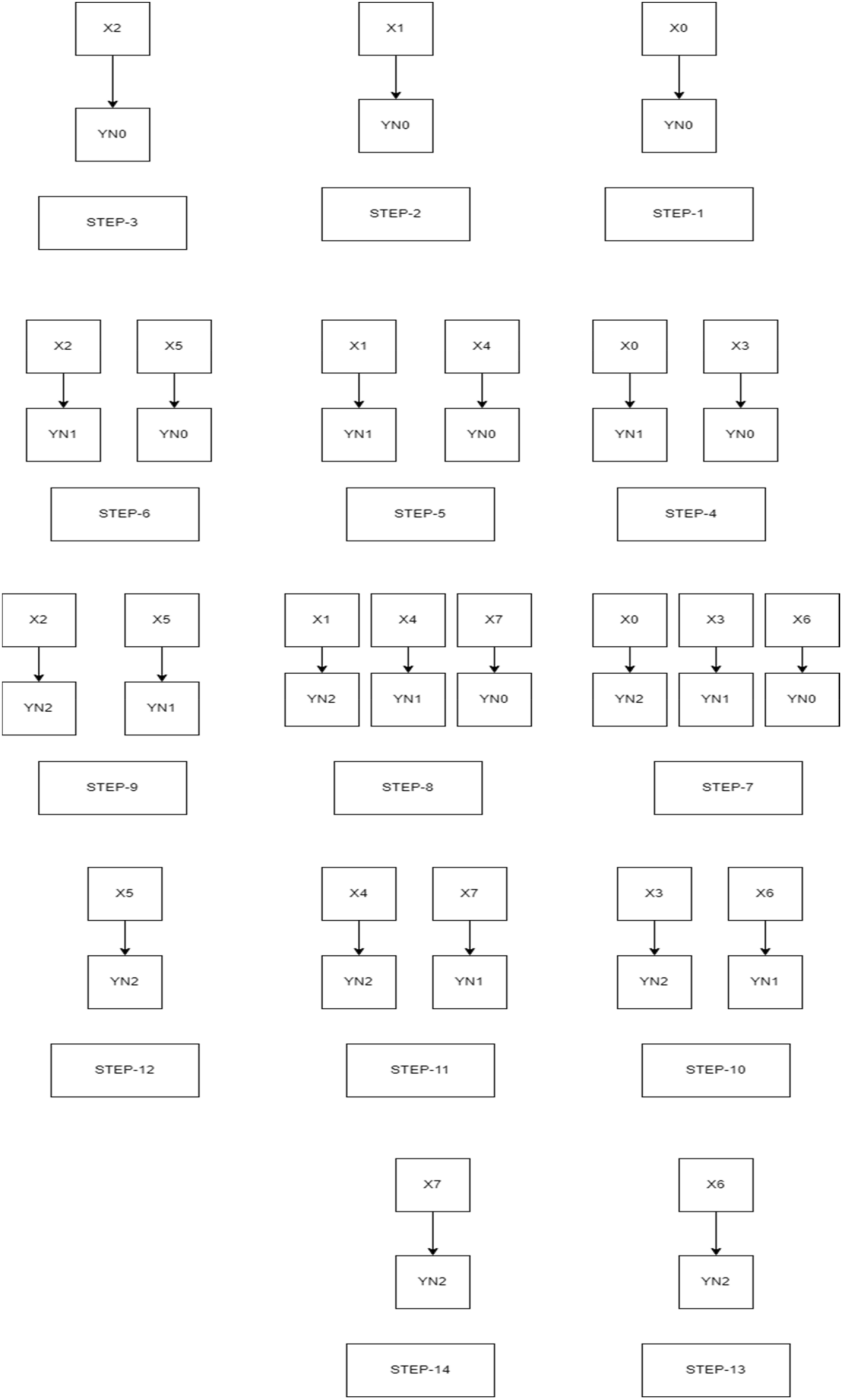
Vedic multiplication of 8-bit-width using criss-cross sutra.

Figure 7 deliberates about the bit-width of 4 × 4 UT sutra, the multiplication operation is accomplished as follows: calculation of the operand was done using a vertical and cross-wise manner with the completion of 6 steps. Similarly, for a bit-width of 8 × 8, the same method is followed by conducting 14 steps, and is shown in Fig. 8. Due to the larger size, of the 16 × 16 multiplier, the criss-cross method was not specified in the paper, but the procedure is the same as the previous method.

After performing the multiplication using UT sutra, the PP rows are generated according to the bit-width of varying operand sizes. By completing the final addition result, it is eventually sent on to the encoder, where the final addition is performed to achieve the 2N-bit product result.

Table 5 shows the encoding rule to be followed after the first and second stages of PPG and PPR are over. These values are to be referred to with the above encoding table after the application of both the algorithms of the Booth and Vedic sutras, and these values are finally fed to the adder to perform the final PPA result.

**Table 5:** Encoded values.

S.no.	PPs	Encoded values
1.	0	0000
2.	1	0001
3.	2	0010
4.	3	0011
5.	4	0100
6.	− 4	1100
7.	− 3	1101
8.	− 2	1110
9.	− 1	1111

### Stage-III-PPA-addition

#### BVR-4-PPA

**Figure 9:**
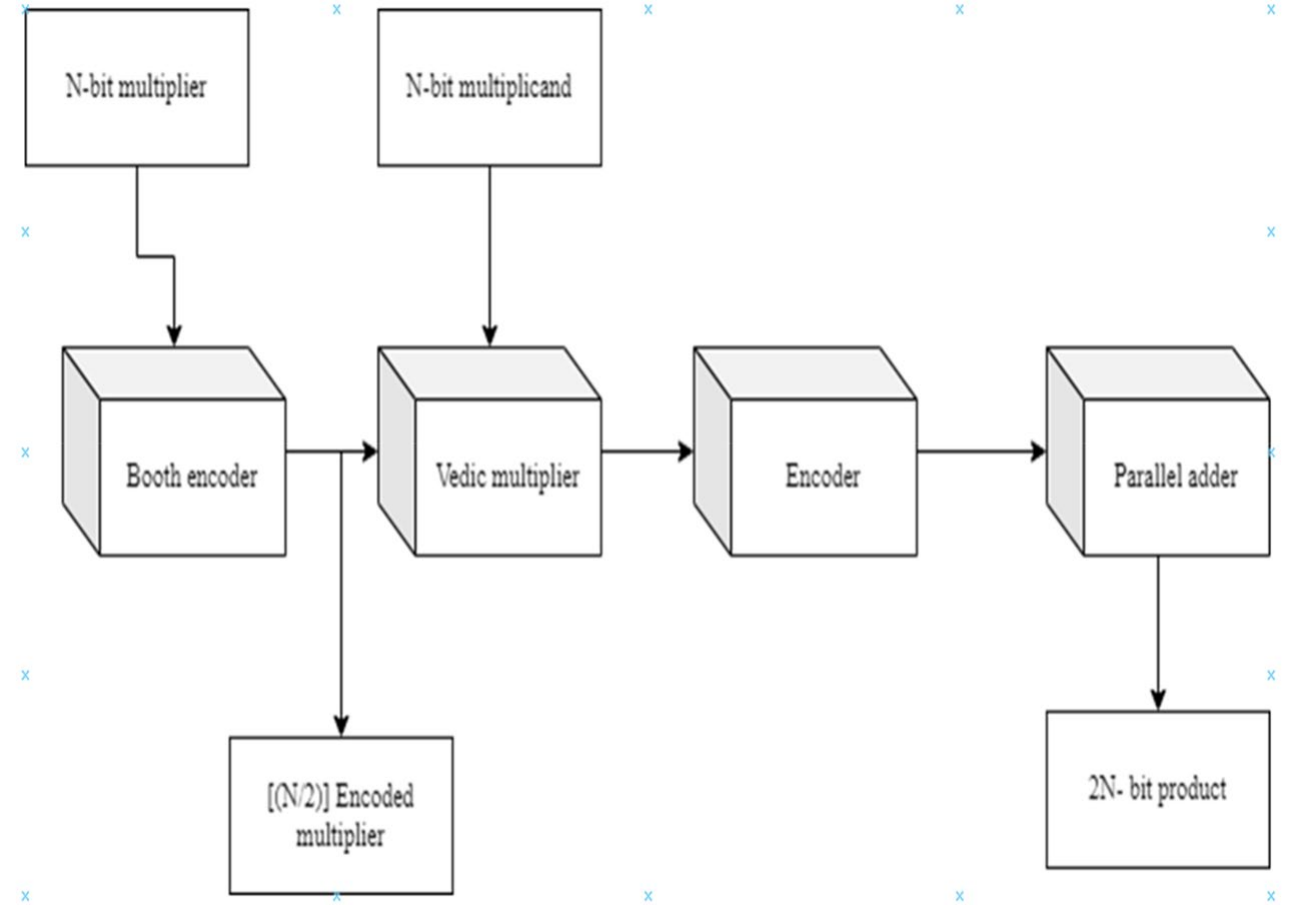
Proposed architecture for BVR-4.

As the multiplier bits are sent to the Booth encoding, which executes the Radix-4 encoding procedure in Fig. 9, the PP rows are cut in half when compared to the usual technique. Following the Radix-4 Booth encoding process, the multiplicand bits conduct Vedic multiplication of UT (criss-cross multiplication) with the new encoded groups and then passed onto the encoder to obtain the final 2N-bit-width result which is achieved by the parallel adder.

**Figure 10:**
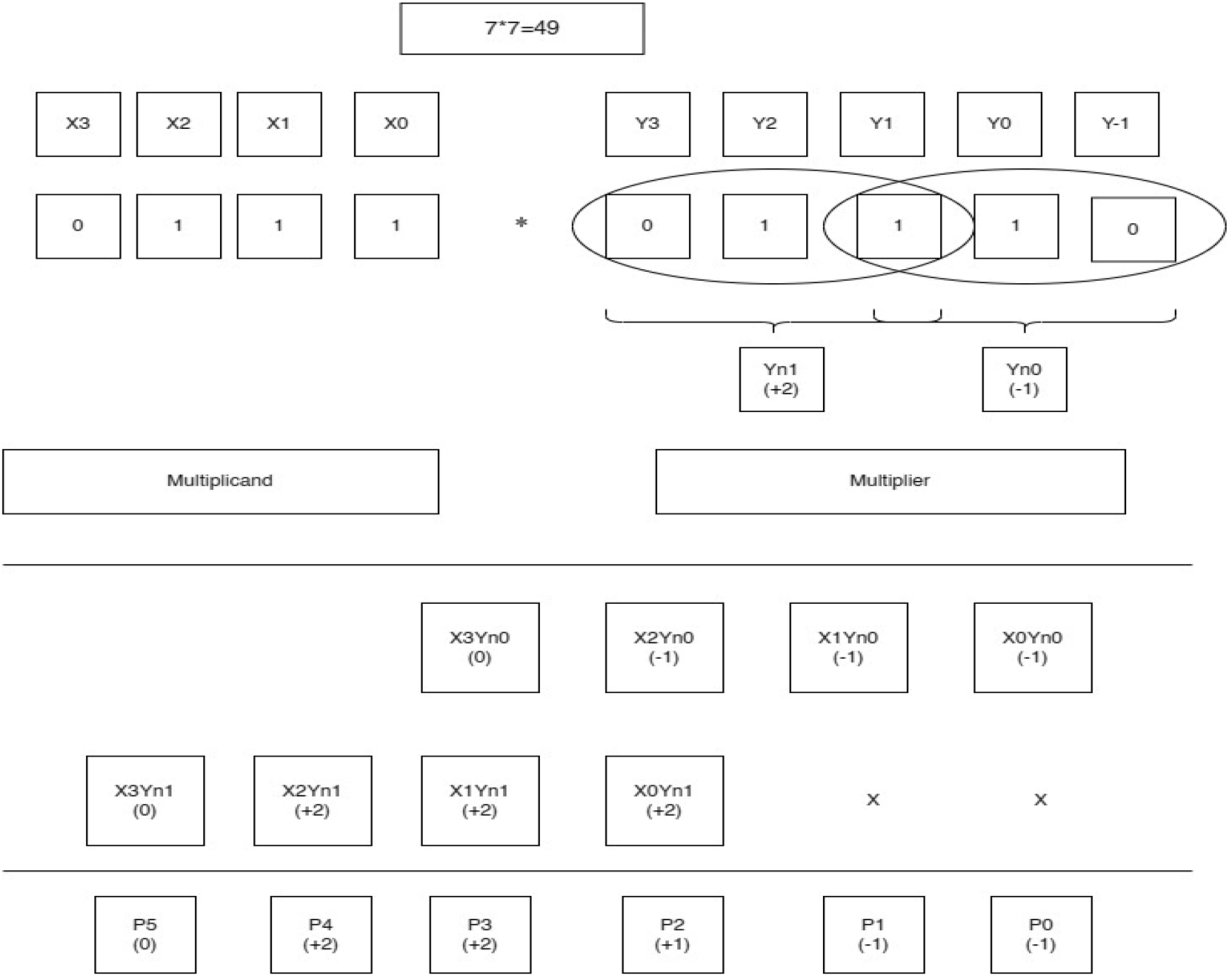
Worked out an example for BVR-4 of bit-width 4. P0 = X0Yn0, P1 = X1Yn0, P2 = X2Yn0 + X0Yn1, P3 = X3Yn0 + X1Yn1, P4 = X2Yn1, P5 = X3Yn1, Final Product = P0 + P1 + P2 + P3 + P4 + P5.

**Figure 11:**
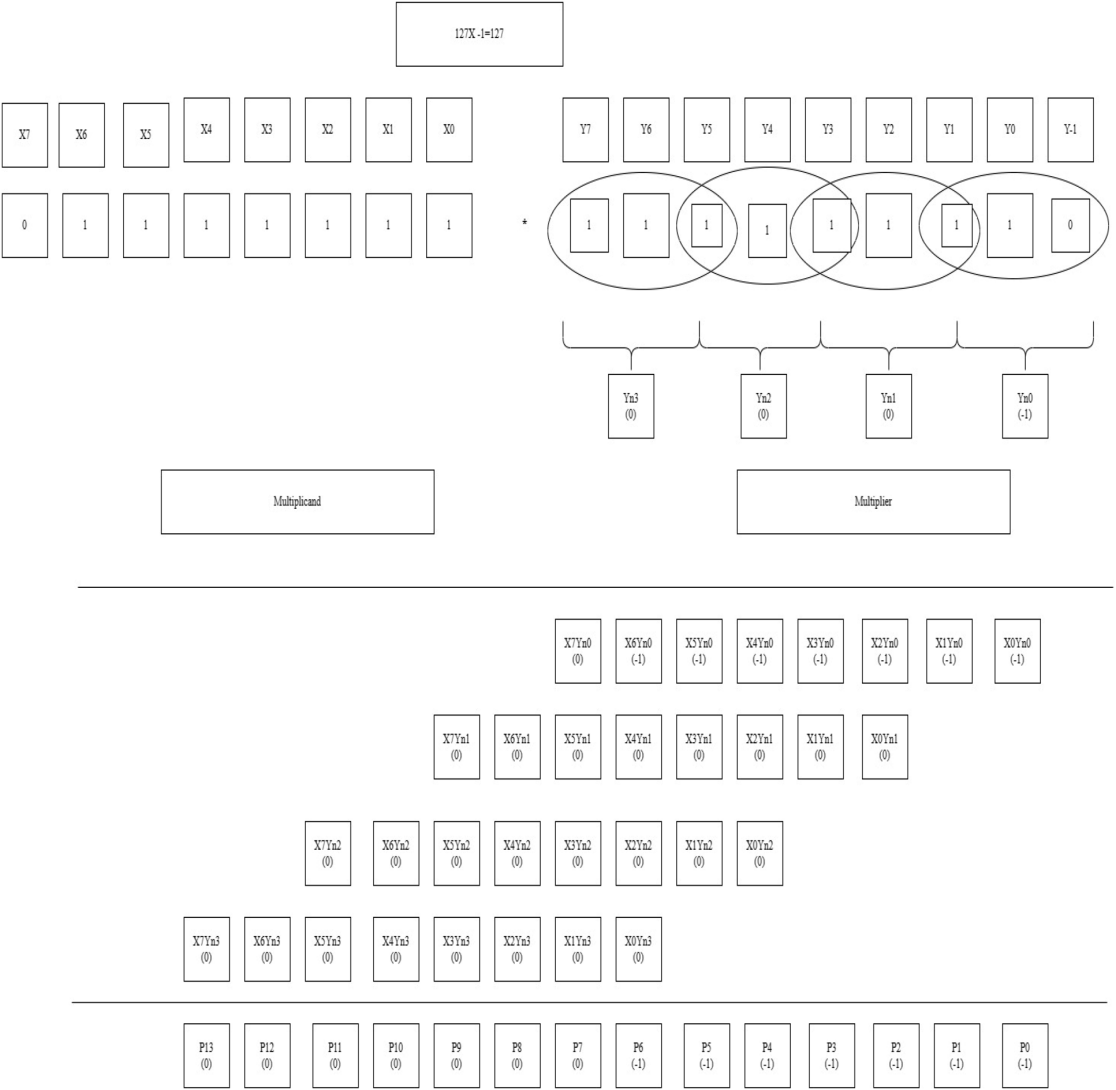
Worked out an example for BVR-4 of bit-width 8. P0 = x0yn0, P1 = x1yn0, P2 = x2yn0 + x0yn1, P3 = x3yn0 + x1yn1, P4 = x4yn0 + x2yn1 + x0yn2, P5 = x5yn0 + x3yn1 + x1yn2, P6 = x6yn0 + x4yn1 + x2yn2 + x0yn3, P7 = x7yn0 + x5yn1 + x3yn2 + x1yn3, P8 = x6yn1 + x4yn2 + x2yn3, P9 = x7yn1 + x5yn2 + x3yn3, P10 = x6yn2 + x4yn3, P11 = x7yn2 + x5yn3, P12 = x6yn3, P13 = x7yn3.

One of the examples of the BVR-4 algorithm is mentioned below. The example shown in Fig. 10 is for operand size 4 × 4. The multiplicand values are assigned as X0, X1, X2, X3, and the multiplier values are assigned as Y-1, Y0, Y1, Y2, Y3. After assigning the values for both the multiplicand and the multiplier, a “0” is added at the LSB of the multiplier bits, then the multiplier bits are grouped into trits and the process is followed for contiguous trits of successive bits. The newly encoded groups are formed as Yn0, and Yn1, and then, by following the values in Table 3, the multiplication operation is carried out to form the PP rows of 1 and 2. After the generation of the first PP row, the successive row is shifted by 2-bit values. Those rows are added and form an intermediate result as P0, P1, P2, P3, P4, P5. The relevant encoded values for the obtained partial values in Fig. 10 are to be referred to in Table 5. The final product is obtained using a sign-magnitude representation of encoded values. The following procedure is carried out for varying operand sizes of 8, 16, etc., and shown in Fig. 11.

By the completion of both the stages of PPG and PPR, the encoded bits obtained in the PPR stage are fed to the encoder and each intermediate resultant is sign extended up to 8 bits and added to acquire the final product shown in Table 6.

**Table 6:** Sign extension of 8-bits of Radix-4.

8	7	6	5	4	3	2	1	0	PP
1	1	1	1	1	**1**	**1**	**1**	**1**	P0
1	1	1	1	**1**	**1**	**1**	**1**	–	P1
0	0	0	**0**	**0**	**0**	**1**	–	–	P2
0	0	**0**	**0**	**1**	**0**	–	–	–	P3
0	**0**	**0**	**1**	**0**	–	–	–	–	P4
**0**	**0**	**0**	**0**	–	–	–	–	–	P5
0	0	0	1	1	0	0	0	1	Final product

Significant values are in bold.

By the completion of both the stages of PPG and PPR, the encoded bits obtained in the PPR stage are fed to the encoder and each intermediate resultant is sign extended up to 16 bits and added to acquire the final product shown in Table 7.

**Table 7:**
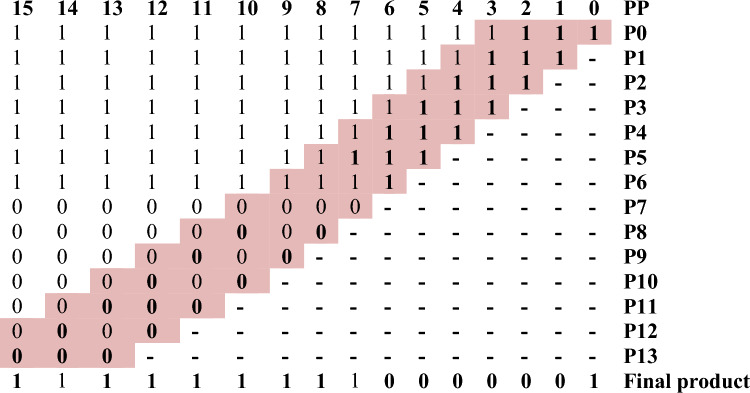
Sign extension of 16-bits of Radix-4.

Significant values are in bold.

The colored (highlighted) digits represent the encoded value according to the Radix-4 and Radix-8 rules.

#### BVR-8-PPA

**Figure 12:**
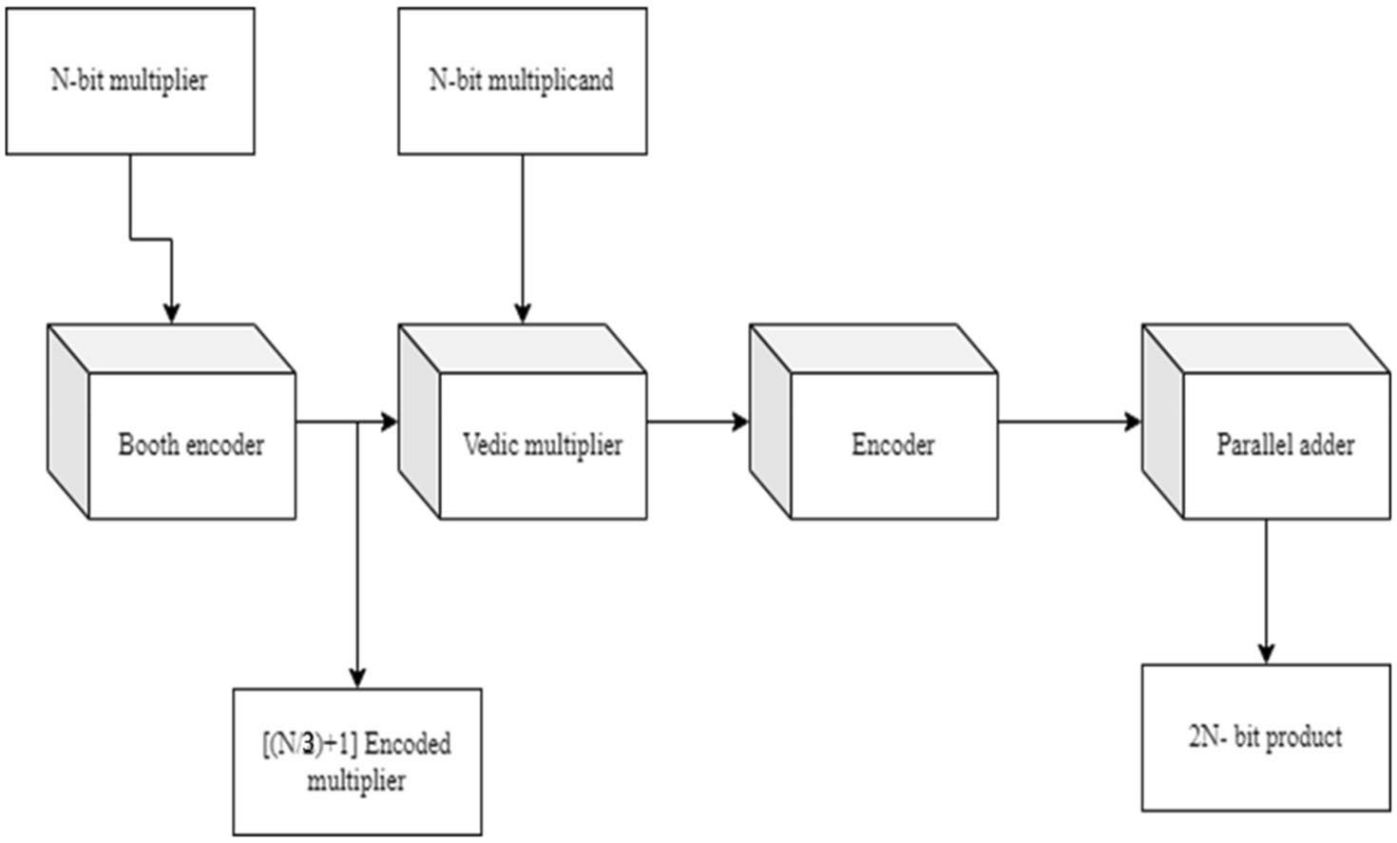
Proposed architecture for BVR-8.

When the multiplier bits are assigned to the booth encoding, which conducts the Radix-8 encoding process, the PP rows are reduced from N to (N/3) + 1 when compared to the usual technique. When compared to the Radix-4 Booth approach, the PP rows are reduced even further in this encoding process. After completing the Radix-8 Booth encoding procedure, the multiplicand bits conduct Vedic multiplication of UT (criss-cross multiplication) with the newly encoded groups and then passed onto the encoder to obtain the final 2N-bit-width result, which is achieved by the parallel adder and it is shown in Fig. 12.

**Figure 13:**
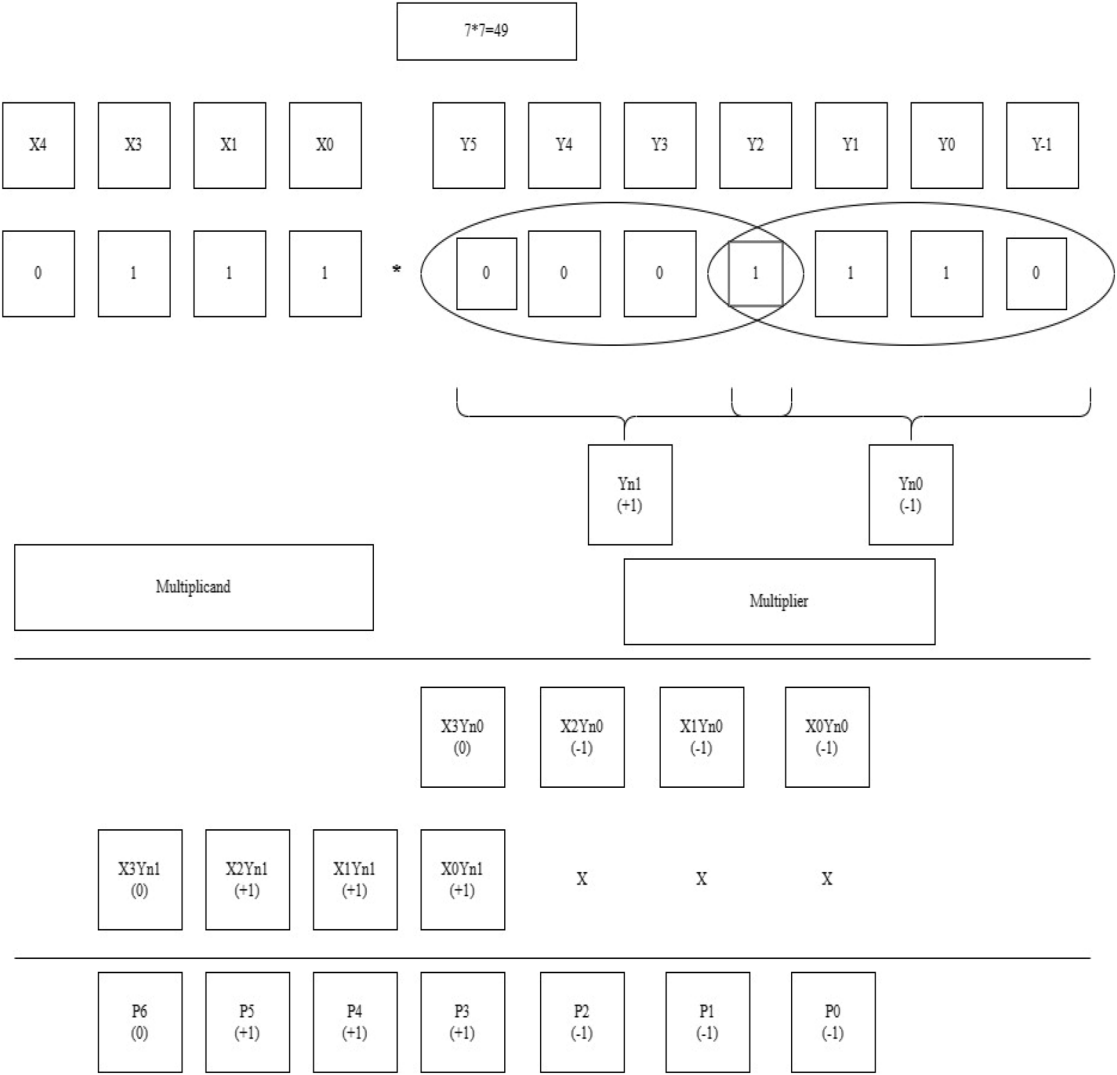
Worked out an example for BVR-8 of bit-width 4. P0 = X0Yn0, P1 = X1Yn0, P2 = X2Yn0, P3 = X3Yn0 + X0Yn1, P4 = X1Yn1, P5 = X2Yn1, P6 = X3Yn1, Final Product = P0 + P1 + P2 + P3 + P4 + P5 + P6.

**Figure 14:**
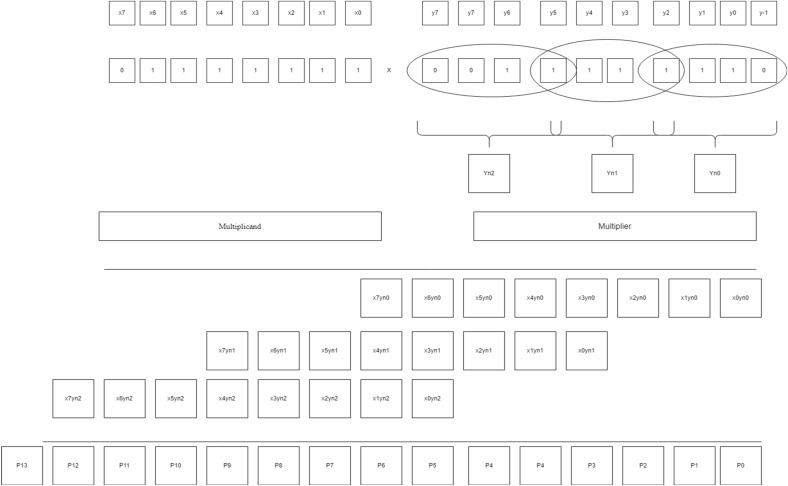
Worked out an example for BVR-8 of bit-width 8. P0 = x0yn0, P1 = x1yn0, P2 = x2yn0, P3 = x3yn0 + x0yn1, P4 = x4yn0 + x1yn1, P5 = x5yn0 + x2yn1, P6 = x6yn0 + x3yn1 + x0yn2, P7 = x7yn0 + x4yn1 + x1yn2, P8 = x5yn1 + x2yn2, P9 = x6yn1 + x3yn2, P10 = x7yn1 + x4yn2, P11 = x5yn2, P12 = x6yn2, P13 = x7yn2.

One of the examples of the BVR-8 algorithm is mentioned below. The example shown in Fig. 13 is for operand size 4 × 4. The multiplicand values are assigned as X0, X1, X2, X3, and the multiplier values are assigned as Y-1, Y0, Y1, Y2, Y3, Y4 and Y5. After assigning the values for both the multiplicand and the multiplier, a “0” is added at the LSB of the multiplier bits, then the multiplier bits are grouped into quads and the process is followed for contiguous quads of successive bits. The newly encoded groups are formed as Yn0, and Yn1, and then, by following the values in Table 4, the multiplication operation is carried out to form the PP rows of 1 and 2. After the generation of the first PP row, the successive row is shifted by 3-bit values. Those rows are added and form an intermediate result as P0, P1, P2, P3, P4, P5 and P6. The relevant encoded values for the obtained partial values in Fig. 13 are to be referred to in Table 5. The final product is obtained employing a sign-magnitude representation of encoded values. The following procedure is carried out for varying operand sizes of 8, 16, etc., and shown in Fig. 14.

By the completion of both the stages of PPG and PPR, the encoded bits obtained in the PPR stage are fed to the encoder and each intermediate resultant is sign extended up to 8 bits and added to acquire the final product shown in Table 8.

**Table 8:** Sign extension of 8-bits of Radix-8.

8	7	6	5	4	3	2	1	0	PP
1	1	1	1	1	1	**1**	**1**	**1**	P0
1	1	1	1	1	**1**	**1**	**1**		P1
1	1	1	1	**1**	**1**	**1**			P2
0	0	0	**0**	**0**	**1**				P3
0	0	**0**	**0**	**1**					P4
0	**0**	**0**	**1**						P5
**0**	**0**	**0**							P6
0	0	0	1	1	0	0	0	1	Final product

Significant values are in bold.

By the completion of both the stages of PPG and PPR, the encoded bits obtained in the PPR stage are fed to the encoder and each intermediate resultant is sign extended up to 16 bits and added to acquire the final product shown in Table 9.

**Table 9:**
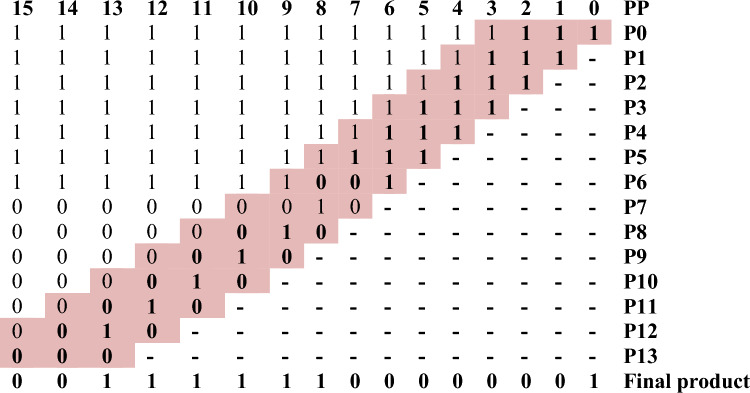
Sign extension of 16-bits.

Significant values are in bold.

The colored (highlighted) digits represent the encoded value according to the Radix-4 and Radix-8 rules.

## Results and discussion

The design of the proposed BVR-4 and BVR-8 methods consists of design entry, synthesis, simulation, and implementation in various FPGA devices and ASIC tools. The proposed architecture has been coded in Verilog language. When there is no error, then the code is synthesized using the Xilinx tool. The suggested methods mathematical model and algorithm are run and verified using the Xilinx Vivado tool. The simulation results demonstrate that the mathematical model can multiply two signed integers. The suggested architectures of Proposed BVR-4 and BVR-8 functional verification are carried out by creating the architecture in Verilog HDL and simulating it in ISIM RTL Simulator. The HDL code is generated in two platforms, Vivado 2019.1 Xilinx Synthesis Technology of FPGA platform and ASIC TSMC 45 nm standard cell typical libraries.

### FPGA implementation

In FPGAs, a look-up-table (LUT) is a small asynchronous SRAMs that is used to implement combinational logic circuits, while flip-flops are single-bit memory cells that are used to hold state. Table 10 shows the comparison of Floating Point, Booth, and Vedic multiplier with the proposed methods, and it is implemented with the Xilinx Vivado Artix-7 XC7A100T-CSG324 device specification. The proposed architecture of BVR-4 is compared with the various multiplier techniques and shows a LUT (Look-up-table) and ADP improvement of 42%, 20% for a bit-width of 4, for a bit-width of 8 it is 94% and 84%, and for 16-bit width range it is 96% and 90%. The proposed architecture of BVR-8 is compared with the various multiplier techniques and shows a LUT (Look-up-table) and ADP improvement of 89%, 83% for a bit-width of 4, for a bit-width of 8 it is 94% and 90%, and for 16-bit width range it is 89% and 72% respectively. The area is calculated in terms of LUTs, Flip-flops, and configurable logic blocks. This table shows a clear view that the proposed method of BVR-4 and BVR-8 is very efficient when we combine the techniques of both Booth and Vedic multiplier concepts.

**Table 10:** FPGA synthesis results.

Number of bit-width	Multipliers	LUTs	Delay	ADP
4	Floating point multiplier^45^	19	4.1	77.9
	Booth multiplier^46^	16	4.1	65.6
	Vedic multiplier^47^	16	4.6	73.6
	Proposed method (Booth Vedic Radix-4)	**11**	5.363	**61.996**
	Proposed method (Booth Vedic Radix-8)	**2**	6.461	**12.922**
8	Floating point multiplier^45^	118	4.7	554.6
	Booth multiplier^46^	101	4.7	474.4
	Vedic multiplier^47^	71	4.7	333.7
	Proposed method (Booth Vedic Radix-4)	**7**	12.59	**88.172**
	Proposed method (Booth Vedic Radix-8)	**5**	10.554	**52.77**
16	Floating point multiplier^45^	548	6.3	3452.4
	Booth multiplier^46^	455	6.3	2866.5
	Vedic multiplier^47^	294	7.6	2234.4
	Proposed method (Booth Vedic Radix-4)	**18**	18.57	**334.314**
	Proposed method (Booth Vedic Radix-8)	**59**	15.83	**934.383**

Significant values are in bold.

**Figure 15:**
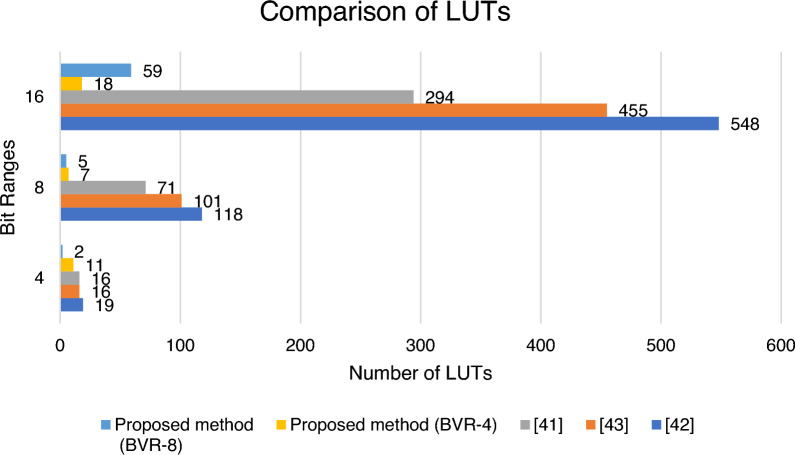
Performance of LUTs for BVR-4 and BVR-8.

Figure 15 shows the graphical representation of the performance ratings of the LUTs with the existing multiplier. However, when all the parameters are considered together, it is evident from the graph that proposed BVR-4 improves for higher bit-widths and BVR-8 shows a better improvement for smaller bit-widths. It has a significant reduction in LUTs compared to existing multiplier architecture.

**Figure 16:**
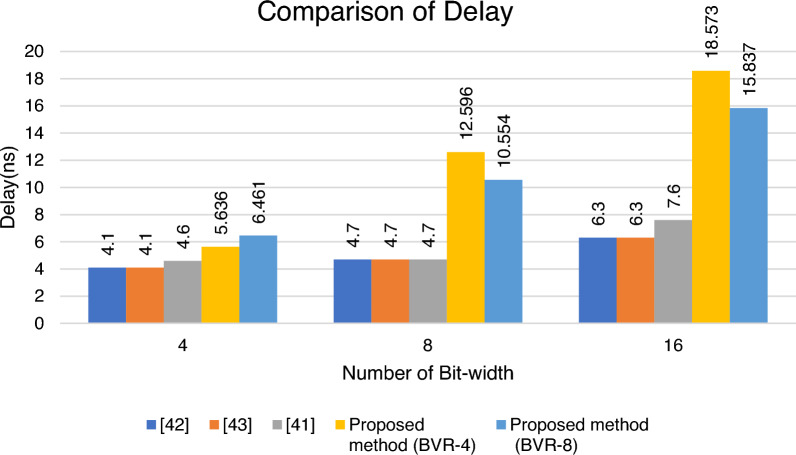
Analysis of delay for BVR-4 and BVR-8.

Figure 16 shows that the delay was decreased when compared with the prior state-of-the-art multipliers. It shows the comparison of the delay for the proposed BVR-4 and BVR-8 with the existing multipliers.

**Figure 17:**
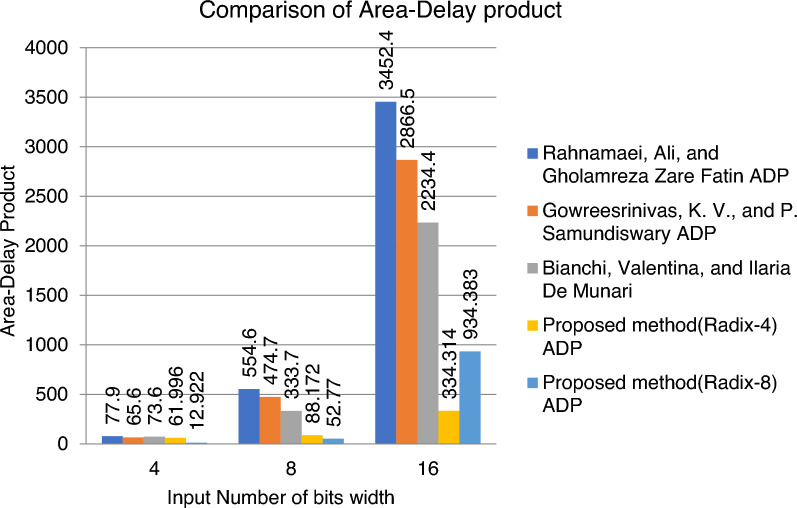
Comparison of Area-Delay Product for the proposed method of BVR-4 and BVR-8.

All the architectures, exploiting the different number of bits for the operands and three different architectures, are compared with the proposed method of BVR-4 and BVR-8 described for the techniques used and analyzed the Area-Delay product it is shown in Fig. 17.

### ASIC implementation

The proposed architectures are realized in the ASIC environment to evaluate the efficiency of the design in terms of the VLSI parameters like area, power, delay, Area Delay Product (ADP), and power delay-product (PDP). A tool Command Language (TCL) script is written to automate the synthesis process of the Verilog HDL code in Mentor Graphics. The design is synthesized for different operand sizes 4, 8, and 16 with TSMC 45 nm standard cell typical libraries. The same process is repeated for the state-of-the-art multipliers considered for the comparison, and the results are given in Table 11.

**Table 11:** Performance comparison of BVR-4 and BVR-8.

N	Multipliers	Area (µm^2^)	Power (µW)	Delay (ns)	Area-delay product (ADP)-pm^2^*s	Power-delay product (PDP)-pJ
4	CSA architecture^48^	114	11	0.8	0.09	0.008
	Parallel-prefix architecture^48^	140	12	0.8	0.1	0.009
	Proposed BVR-4	88	10.2	0.4	0.035	0.004
	Proposed BVR-8	58	9.5	0.3	0.001	0.002
8	CSA architecture^48^	452	35	2.1	0.97	0.07
	Parallel-prefix architecture^48^	587	40	0.8	0.46	0.03
	Proposed BVR-4	322	32	0.8	0.25	0.02
	Proposed BVR-8	291	30.23	0.75	0.21	0.026
16	CSA architecture^48^	2030	208	9.5	19.18	1.976
	Parallel-prefix architecture^48^	2679	260	6.0	16.07	1.56
	Proposed BVR-4	1176	205	1.5	1.76	0.03
	Proposed BVR-8	1123	200	1.4	1.577	0.02

Table 11 shows that the Proposed BVR-4 and BVR-8 multiplier architecture outperforms in all three aspects followed by traditionally signed CSA and parallel prefix architecture because of less area, increased speed with reduced power. If the number of transistors is optimized, it offers a better output of the proposed methods of BVR-4 and BVR-8. So, 45 nm technology is significantly preferable to reduce area, power, and delay. In 45 nm BVR-4 architecture contains area improvement of 22%, power improvement of 7%, and speed improvement of 50% for the bit-width of 4. While for the bit-width of 8, it shows an area improvement of 28%, power improvement of 8%, and speed improvement of 61% and for the operand size of 16 it shows an area improvement of 42%, power improvement of 1% and speed improvement of 84% when compared with the CSA architecture. The proposed BVR-8 architecture contains an area improvement of 49%, power improvement of 13%, and speed improvement of 62% for the bit-width of 4, while for the bit-width of 8, it shows an area improvement of 35%, power improvement of 13% and speed improvement of 64%, for the operand size of 16 it shows an area improvement of 44%, power improvement of 3% and speed improvement of 85%.

**Figure 18:**
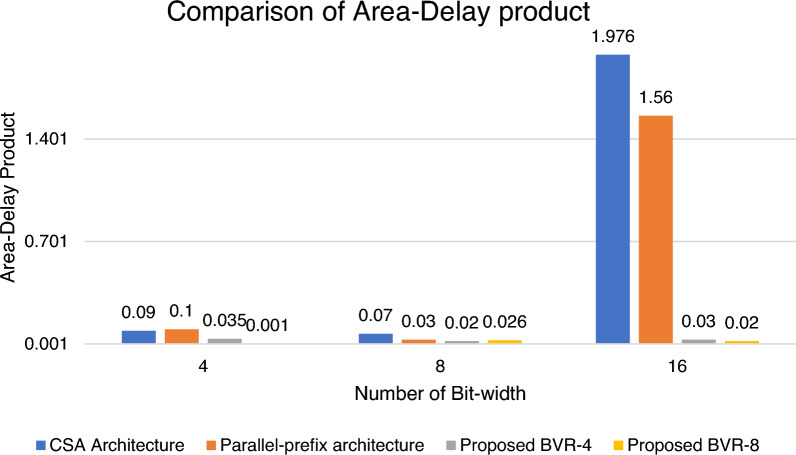
Comparison of Area-Delay Product with existing multipliers.

Figure 18 shows the BVR-4 architecture contains 0.035, 0.25 and 1.76 of Area-Delay Product for the Bit-width range of 4, 8 and 16 when compared with the previous CSA and parallel prefix architecture in 45 nm ASIC standard cell libraries. For BVR-8 architecture it contains 0.001, 0.21, and 1.577 for the operand size of 4, 8 and 16.

**Figure 19:**
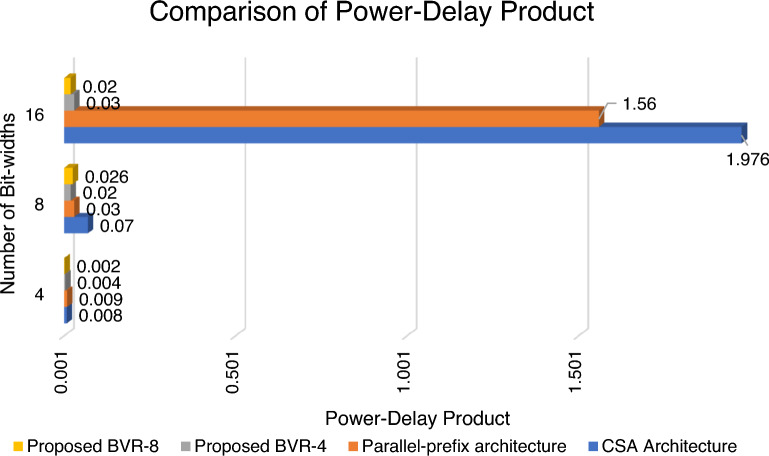
Comparison of power-delay product of proposed BVR-4 and BVR-8.

Figure 19 shows the power-delay product comparison of BVR-4 and BVR-8 with the existing CSA and parallel prefix architecture. It shows a PDP of 0.04, 0.02, and 0.03 for the BVR-4 method, and for BVR-8 it shows 0.002, 0.026, and 0.02 respectively.

## Conclusion

In this paper, a new method of signed digit multiplication is presented. The proposed design is based on both the techniques of Booth and the Vedic multiplier concept using the sutra of Urdhva-tiryakbhyam (criss-cross) multiplication technique. First, the scope of the booth multiplier is extended to the Vedic multiplication to perform parallel processing at the stage of partial product reduction stage, which leads to a decreased propagation delay. The propagation delay issue is resolved using an adder and in sign-magnitude representation. The proposed design is found to have a high speed with minimal area consumption with various state-of-the-art architectures. For Booth-Vedic-Radix-4 encoding (BVR-4) decreases area by 89% and improves area-delay product (ADP) by 72% for a 16-bit multiplier when subjected to the Conventional Radix-4 booth multiplier of different operand sizes. The Booth-Vedic-Radix-8 (BVR-8) method shows that there will be an 89% reduction in area and improves ADP by 72% for the 16-bit multiplier. This work can be further incorporated into the image compression technique to achieve the rapid result.

## Data Availability

The data analyzed during the current study available from the corresponding author on reasonable request.
